# Isolation and Characterization of a Novel Cyanophage Encoding Multiple Auxiliary Metabolic Genes

**DOI:** 10.3390/v14050887

**Published:** 2022-04-24

**Authors:** Cuhuang Rong, Kun Zhou, Shuiming Li, Kang Xiao, Ying Xu, Rui Zhang, Yunlan Yang, Yu Zhang

**Affiliations:** 1 Shenzhen Key Laboratory of Marine Bioresource and Eco-Environmental Science, Guangdong Engineering Research Center for Marine Algal Biotechnology, College of Life Sciences and Oceanography, Shenzhen University, Shenzhen 518060, China; rongcuhuang1@email.szu.edu.cn (C.R.); shuimingli@szu.edu.cn (S.L.); xiaokang@ustc.edu (K.X.); boxuying@szu.edu.cn (Y.X.); 2 Department of Ocean Science, The Hong Kong University of Science and Technology, Hong Kong 999077, China; kzhouad@connect.ust.hk; 3 State Key Laboratory of Marine Environmental Science, Fujian Key Laboratory of Marine Carbon Sequestration, College of Ocean and Earth Sciences, Xiamen University, Xiamen 361102, China; ruizhang@xmu.edu.cn

**Keywords:** cyanophage, genome, auxiliary metabolic genes, proteome

## Abstract

As significant drivers of cyanobacteria mortality, cyanophages have been known to regulate the population dynamics, metabolic activities, and community structure of this most important marine autotrophic picoplankton and, therefore, influence the global primary production and biogeochemical cycle in aquatic ecosystems. In the present study, a lytic *Synechococcus* phage, namely S-SZBM1, was isolated and identified. Cyanophage S-SZBM1 has a double-stranded DNA genome of 177,834 bp with a G+C content of 43.31% and contains a total of 218 predicted ORFs and six tRNA genes. Phylogenetic analysis and nucleotide-based intergenomic similarity suggested that cyanophage S-SZBM1 belongs to a new genus under the family *Kyanoviridae*. A variety of auxiliary metabolic genes (AMGs) that have been proved or speculated to relate to photosynthesis, carbon metabolism, nucleotide synthesis and metabolism, cell protection, and other cell metabolism were identified in cyanophage S-SZBM1 genome and may affect host processes during infection. In addition, 24 of 32 predicted structural proteins were identified by a high-throughput proteome analysis which were potentially involved in the assembly processes of virion. The genomic and proteomic analysis features of cyanophage S-SZBM1 offer a valuable insight into the interactions between cyanophages and their hosts during infection.

## 1. Introduction

Marine picocyanobacteria consisting of *Synechococcus* and *Prochlorococcus* are the most abundant and geographically widespread photosynthetic organisms in the oceans [1]. With a global mean annual abundance of 3.6 × 10^27^ cells, picocyanobacteria account for ~15% of marine bacterial biomass and are responsible for ~25% of oceanic CO_2_ fixation [2,3,4]. It has been reported that up to 5% of marine cyanobacterial cells contain mature cyanophage particles [5]. Lysis mediated by cyanophages results in the release of cellular dissolved organic matter and, therefore, influences the community structure, primary production, and global biogeochemical cycles [6,7]. Cyanophages also impact the genomic diversity and evolution of their hosts through horizontal gene transfer and antagonistic coevolution [8,9,10].

Owing to the increasing availability of viral metagenomes, the underlying structure of global viral communities has been gradually revealed [11,12]. However, genome-level viral diversity and virus–host interactions cannot be thoroughly explored in virome studies. As such, the physiological and genomic characterizations of isolated viruses, especially those that infect dominant bacterial groups, will provide important opportunities to advance the understanding of viral-induced cellular mechanisms and their biogeochemical impact. As representative systems for virus–host interactions research, cyanophages generally encode auxiliary metabolic genes (AMGs) which can influence the metabolism of infected cells by expressing AMGs and are thought to enhance cyanophages fitness in marine environments [13,14,15]. To date, cyanophage-encoded AMGs are always involved in photosynthesis, carbon metabolism, cycling of nutrients (such as nitrogen, phosphorus, and sulfur), nucleic acid synthesis and metabolism, and stress tolerance [16,17,18,19,20]. However, only the most essential AMGs are likely to be maintained in phage genomes on account of high cost; therefore, the genomic content of AMGs in cyanophages may provide important clues to environmental selection and evolution [15,21].

In this study, we reported the discovery and characterization of a novel cyanophage S-SZBM1, which infects *Synechococcus* CB0101. The host used in this study was isolated from the Chesapeake estuary and has been deeply explored regarding its distribution, genome features, and responses to environmental stresses [22,23,24]. Therefore, *Synechococcus* CB0101 was thought to be a good model strain for phage isolation and the investigation of phage–host interactions. To explore the potential functional linkage between cyanophage and its host, we sequenced the genome of cyanophage S-SZBM1 and identified the major structural proteins. We expect that systematic isolation and characterization of cyanophages will provide new insights into phage–host interactions in terms of their biological features, genetics, and proteomics.

## 2. Materials and Methods

### 2.1. Cyanophage Isolation, Purification, and Enrichment

As the bacterial host, *Synechococcus* CB0101 was grown in SN medium with 15‰ sea salt (SN15) at 22–25 °C under cool white light of 20–30 μEm^−2^s^−1^ [25,26]. Virus-containing surface water was collected from Shenzhen Bay, Guangdong, China in March 2017 and used for phage isolation by the double-layer agar method under the host-culture conditions [20,27]. Cyanophage was picked from an individual plaque at least five times and co-cultivated in liquid SN15 medium with the host for expansion. High-titer phage suspensions for morphological observation and DNA extraction were prepared by the method of cesium chloride (CsCl) density gradient ultracentrifugation [28]. In brief, phages were co-cultured with host until cyanobacterial lysis occurred, and the lysate was filtered through 0.22 μm pore size filters to remove cell debris and concentrated by precipitation with polyethylene glycol 8000 (10,000× g, 4 °C, 1 h), followed by CsCl purification (gradient density: 1.5 g·mL^−1^ and 1.7 g·mL^−1^, 200,000× g, 4 °C, 24 h). The purified high-titer phages were desalted and kept in SM buffer (100 mmol·L^−1^ NaCl, 8 mmol·L^−1^ MgSO_4_, 50 mmol·L^−1^ Tris-HCl at pH 7.5) for later analysis.

### 2.2. Specificity Test

The experimental cyanobacteria strains used in the specificity test included 11 marine cyanobacteria strains (*Prochlorococcus marinus* NATL2A, *Synechococcus* CBW1002, CBW1004, CBW1005, CBW1006, CBW1107, CBW1108, WH7803, WH8102, WH8108, XM05) and six freshwater cyanobacteria strains (*Chroococcus* FACHB-193, *Microcystis aeruginosa*, *M. flos-aquae*, *M. ichthyoblabe*, *Synechococcus* PCC 7942, *S. elongatus*). About 10 µL purified phages (filtered through 0.22 μm membrane and the concentration higher than 10^6^ PFU mL^−1^) were added into the exponentially growing cultures (200 µL each well) of cyanobacteria strains in 96-well microtiter plates in triplicate, and then all plates were incubated under the same conditions as described above. The cultures were continuously monitored for a week and compared with the controls visually.

### 2.3. Transmission Electron Microscopic Analysis

The phage morphology was observed by transmission electron microscopy (TEM). Briefly, the purified and desalted phages were added dropwise onto 200-mesh carbon-coated coppers for 10–30 min in the dark. After being negatively stained with 1% phosphotungstic acid and air-dried for 30 min, samples were imaged using a JEM-2100 transmission electron microscope (JEOL, Tokyo, Japan) at 80 kV accelerating voltage. Images were collected using a CCD image transmission system (Gatan, Pleasanton, CA, USA). Capsid size and tail length of phages were determined by measuring at least five phage specimens.

### 2.4. DNA Extraction

Cyanophage genomic DNA was extracted using phenol-chloroform extraction method. The high-titer phages were digested in a lysis solution (20 mg·mL^−1^ proteinase K, 10% SDS, 0.5 mol·L^−1^ EDTA pH 8.0) at 55 °C for 3 h. Then, the phage DNA was extracted with phenol/chloroform/isopentanol (25:24:1), chloroform/isopentanol (24:1), and isopropanol. After rinsing with prechilled 70% ethanol, the genomic DNA was air-dried and then dissolved in TE buffer (10 mmol·L^−1^ Tris-HCl, 1 mmol·L^−1^ EDTA, pH 8.0) and stored at −20 °C before sequencing.

### 2.5. Genomic Analysis

One library with insert size of approximately 300 bp was constructed and sequenced on the Illumina Hiseq4000 platform using HiSeq PE Cluster Kit v4 cBot (Illumina, San Diego, CA, USA). Raw reads were trimmed using Trimmomatic v.0.36 with custom settings (ILLUMINACLIP: TruSeq3-PE.fa:2:30:10 LEADING:3 TRAILING:3 SLIDINGWINDOW:4:15 MINLEN:40) [29]. Then, the reads were assembled using IDBA-UD v.1.1.3 with a list of sequential k-mer sizes from 51 to 87 with a 4-kmer step [30]. A binning approach developed by Albertsen et al. was modified to cluster assembled sequences and to remove cellular DNA sequences that might be derived from bacterial hosts. Sequencing reads associated with non-bacterial bins were extracted using Perl scripts for subsequent reassembly [31]. BBMap (https://sourceforge.net/projects/bbmap/, accessed on 24 July 2018) with the command of bbnorm.sh was used to reduce the sequencing coverage of reads to 100×. Then the normalized reads were corrected and reassembled using SPAdes with a customized kmer list (33, 55, 77, 99) [32]. Finally, only one scaffold >1 kb of the reassembled draft genome was retained. A termini analysis, using a CLC Genomics Workbench (version 3.6.1) with default parameters, was used to identify the phage’s genome termini and genome packaging with high-throughput sequencing data [33]. The open reading frames (ORFs) were predicted by the online server GeneMarkS (version 4.25), and the tRNA sequences were identified by tRNAscan-SE v.2.0 [34]. Translated ORFs were annotated through a BLASTP search against the National Center for Biotechnology Information (NCBI) database with *e*-values ≤ 10^−5^. Genome mapping was visualized based on the genome annotations using Java Operon. For phage taxonomy, vConTACT2 v0.9.19 was employed to compare cyanophage S-SZBM1 gene contents against the ProkaryoticViralRefSeq99 (v99) database with default parameters, and the phages predicted to be related with cyanophage S-SZBM1 were determined by similarity score > 1 [35]. Complete amino acid profiles of cyanophage S-SZBM1 and its related phages were submitted to the virus classification and tree building online resource (VICTOR) (https://ggdc.dsmz.de/victor.php, accessed on 14 July 2021) for phylogenetic analysis, and the recommended settings of genome BLAST distance phylogeny (GBDP) method were used [36]. In addition, intergenomic nucleotide sequence similarity and aligned genome fractions within the imported phages were plotted with Virus Intergenomic Distance Calculator (VIRIDIC) under recommended configuration [37]. The complete genome sequence of cyanophage S-SZBM1 had been submitted to the GenBank database under the accession number OL473597.

### 2.6. Mass Spectrometry-Based Proteomics

Purified virus proteins were extracted from CsCl-purified virus-like particles by placing in a strong lysis buffer (0.1% SDS, 1% Triton X-100, 1 mmol·L^−1^ EDTA, 10 mmol·L^−1^ Tris-HCl pH 7.4) supplemented with protease inhibitor cocktail (Solarbio, Beijing, China). The protein sample was purified and air-dried by acetone precipitation [38,39]. The sample was dissolved in urea solution, while protein content was estimated by an Enhanced BCA Protein Assay Kit (Beyotime, Shanghai, China). Then, total protein sample was reduced in 10 mmol·L^−1^ DTT and alkylated by 40 mmol·L^−1^ iodoacetamide. Next, the samples were diluted in 50 mmol·L^−1^ NH_4_HCO_3_, digested with sequencing grade modified trypsin (Promega, WI, USA) at a final ratio of 1:50 (trypsin: total protein), and trypsin digestion was stopped by adding 0.1% trifluoroacetic acid. Finally, peptides were desalted using ZipTipC18 pipette tips (Millipore, Cork, Ireland) and dried in a Centrivap (Labconco, Kansas City, MO, USA) [40].

### 2.7. NanoLC-ESI-MS/MS Analysis

The lyophilized peptides were re-suspended in LC-MS solvent buffer A (2% acetonitrile containing 0.1% formic acid), then loaded into a nanoLC trap column for trapping and desalting for 5 min with 100% solvent A. Then, a linear elution gradient of 8–38% solvent B (water/acetonitrile/formic acid = 2/98/0.1%) in 30 min was applied on a nano reverse phase HPLC analytical column. An information-dependent acquisition (IDA) model was used to acquire tandem MS data on a Triple TOF 5600 + LC-MS/MS (AB SCIEX, Concord, ON, Canada) fitted with a Nanospray III ion source.

The acquired MS/MS data was converted to Mascot generic format (.mgf) files by ProteinPilot software for further protein identification through the Mascot v.2.3.02 (Matrix Science, London, UK) search engine. Mascot was set up to search the custom composite protein database (base on cyanophage genome annotations) with the following parameters: type of search: MS/MS ion search; enzyme: trypsin; fixed modifications: Carbamidomethyl (C); variable modifications: Acetyl (Protein N-term), Deamidated (NQ), Gln > pyro-Glu (N-term Q), Oxidation (M); peptide mass tolerance: ±30 ppm; fragment mass tolerance: ±0.05Da; max missed cleavages: 1; and the auto-decoy search option was selected. Protein identifications were made at a significance threshold of *p* < 0.05 or target decoy of 1% FDR. Protein lists were exported in csv format for immediate data evaluation and curation to remove contaminants in Excel spreadsheet.

## 3. Results and Discussion

### 3.1. Morphology and Host Range of S-SZBM1

**Figure 1:**
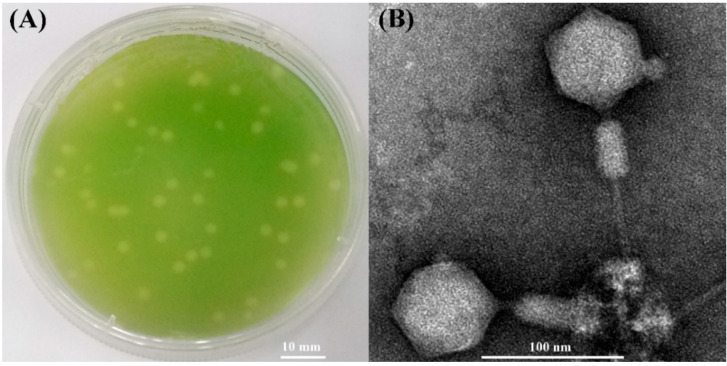
(**A**) Plaques of cyanophage S-SZBM1 formed on the lawn of *Synechococcus* CB0101. (**B**) Transmission electron micrograph of cyanophage S-SZBM1 with non-contracted tail and contracted tail.

A cyanophage named S-SZBM1 was isolated from surface seawater of Shenzhen Bay (22.52° N, 113.96° E), Guangdong, China by infecting *Synechococcus* CB0101. Cyanophage S-SZBM1 formed distinguishable plaques with size ranging from 1.5 to 4.0 mm after a week of growth (Figure 1A). The TEM analysis revealed that cyanophage S-SZBM1 displays myovirus morphology with an isometric and icosahedral head (67.5 ± 1.0 nm in diameter) and a rigid contractile tail (71.7 ± 2.6 – 113.6 ± 4.0 nm long) (Figure 1B). Generally, myoviruses dominated the viral signal in microbial-fraction metagenomic datasets and isolated phages [41,42,43,44]. Among all of the 17 tested freshwater and marine cyanobacteria species/strains, the lytic effect induced by cyanophage S-SZBM1 was only observed in the original host strain. This finding seems to be contrary to previous studies which have suggested a broad host range of myoviruses [45]. For example, myovirus S-CBM2, which was isolated by infecting *Synechococcus* CB0101, can cross-infect several *Synechococcus* strains, including *Synechococcus* WH8101, WH7805, WH7803, and CB0102 [46]. It should be noted that the number and species of tested hosts were limited here, and other yet-to-be-discovered hosts cannot be ruled out.

### 3.2. Genome Features and Taxonomy of S-SZBM1

**Figure 2:**
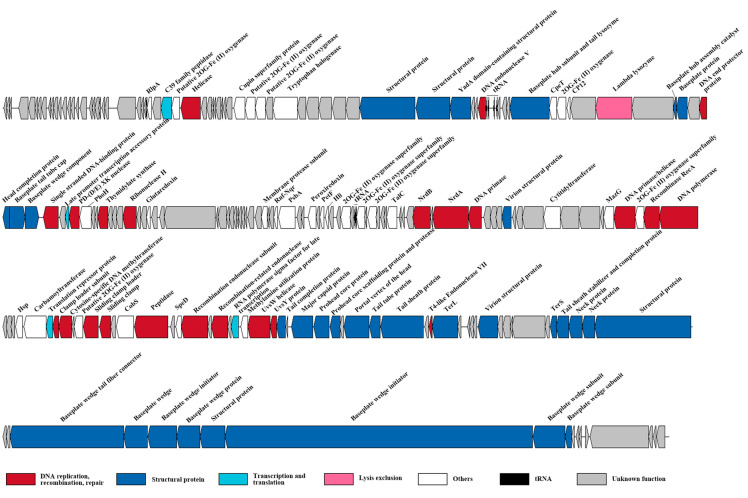
The genome map of cyanophage S-SZBM1. Genes within different functional categories are indicated by colors noted below.

The genome sequencing data indicated that cyanophage S-SZBM1 genome is a 177,834 bp double-stranded, circularly permuted DNA molecule, with a G + C content of 43.31%. A total of 218 predicted ORFs, including 83 conserved domains and accounting for over 95% of the genome size, were detected as probable genes in cyanophage S-SZBM1 (Figure 2, Table S1). The majority of the genes (195 of 218) have plausible homologs in other genomes, with identities of 26.51–95.08%. Among all of the 90 functionally annotated genes, 32 genes were virion structural proteins, 23 genes were involved in DNA replication, recombination, and repair, one gene was viral lysis-related gene, four genes were related to transcription and translation, and 30 genes were annotated as other function. According to the core genes established in previous studies, a pool of 67 T4-like core genes involved in appropriating host metabolic machinery, DNA replication, virion construction, and hypothesis functions, were identified in cyanophage S-SZBM1 genome (Table S2) [14,42,44]. Among these core genes, 51 are in conserved domain. We also identified six tRNA genes in cyanophage S-SZBM1, including two clusters of four genes (Asn, Ala, Pro, Thr) and two genes (Arg, Val) (Figure 2, Table S3).

**Figure 3:**
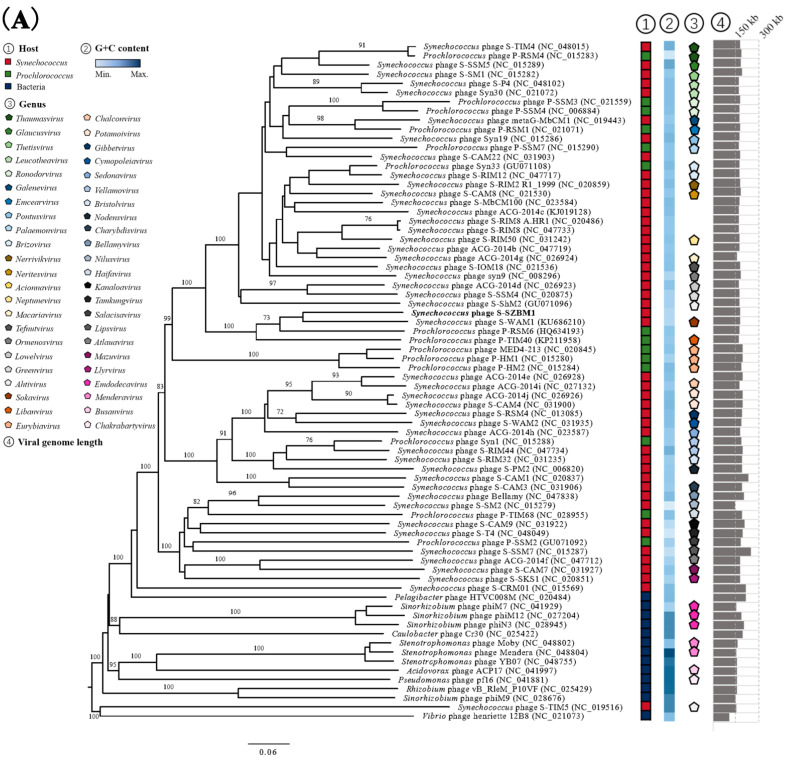

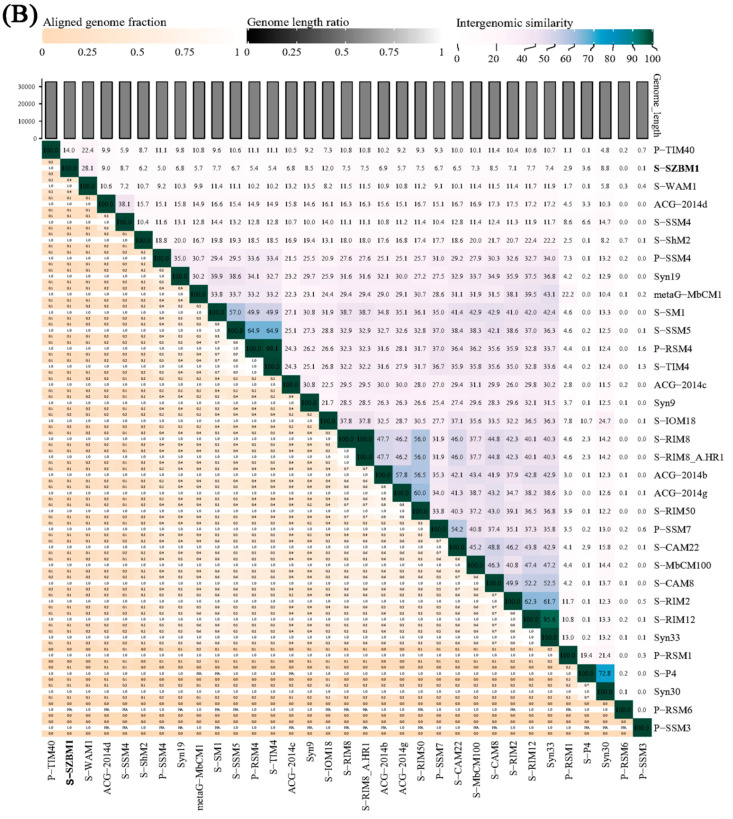
(**A**) Phylogenetic analysis showing the evolutionary relationship and taxonomy of cyanophage S-SZBM1. The GBDP phylogenetic tree was constructed using VICTOR based on complete amino acid profiles of compared phages. The numbers near branches of the phylogenetic tree are GBDP pseudobootstrap support values from 100 replications. The hosts, G+C content, genus cluster information, and genome size of these phages were obtained from the NCBI and the ICTV. (**B**) Heatmap generated by VIRIDIC incorporating intergenomic sequence similarities (right half) and alignment indicators (left half) for cyanophage S-SZBM1 and its most closely related 32 cyanophages.

An initial NCBI BLASTN analysis of the complete nucleotide sequence revealed that cyanophage S-SZBM1 shows limited similarity to 14 cyanophages with low coverages of 2–38% and identities of 74.58–85.49% (Table S4). Meanwhile, a total of 73 phages were predicted to be related with cyanophage S-SZBM1 by vConTACT2, and a GBDP phylogenetic tree was constructed based on amino acid sequences of cyanophage S-SZBM1 and these 73 related phages. In the resulting tree, cyanophage S-SZBM1 is most closely related to three cyanophages, namely S-WAM1, P-RSM6, and P-TIM40 (Figure 3A). In addition, nucleotide-based intergenomic similarities were calculated by VIRIDIC and showed the highest value of only 28.1% between cyanophage S-SZBM1 and S-WAM1 (Figure 3B). According to the guidelines for the demarcation of genus-level rank of phage taxonomy, cyanophage S-SZBM1 should be classified as a representative of an unassigned genus under the family *Kyanoviridae*, while 70% nucleotide identity of the full genome length was established as the cut-off for genera [37,47].

### 3.3. AMGs Encoded by S-SZBM1 Re-Program Phage–Host Interactions

**Figure 4:**
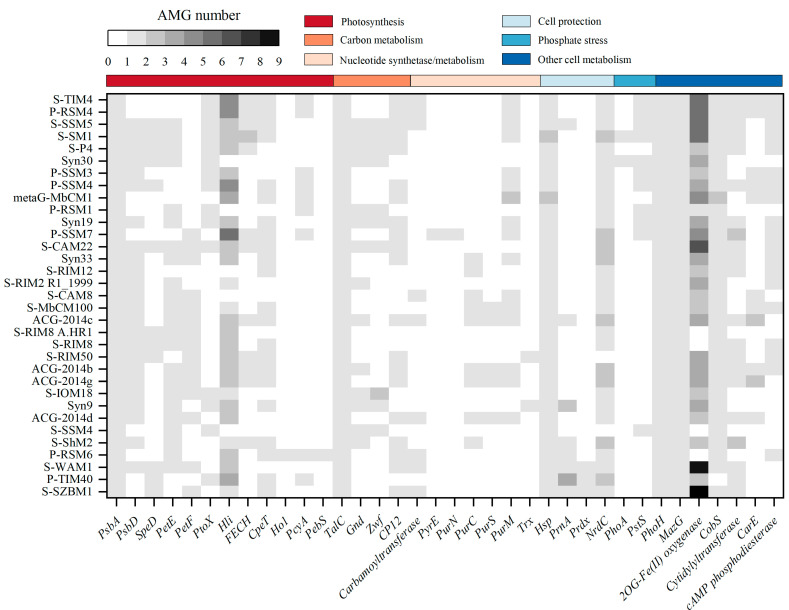
Representative AMGs in cyanophage S-SZBM1 and its most related 32 cyanophages. AMGs within different functional categories are indicated by color noted above.

Previous studies highlighted the prevalence of auxiliary metabolic genes (AMGs) derived from phage genomes and metagenomes, which play important roles in keeping host cell functioning and facilitate replication of phages during infection [18,19,48,49,50]. In other words, phages can re-program the metabolism of their host by introducing AMGs that are involved in energy and carbon metabolism, DNA and RNA processing, and other functions [48]. Among cyanophage S-SZBM1 and its most closely related 32 cyanophages, at least 13 AMGs are presented in a genome, and the frequencies of AMGs range from 3.03% (*hol*, *pyrE*, *purN*) to 100% (*psbA*, *talC*, *mazG*, *phoH*, and *hsp*) (Figure 4). A total of 25 ORFs in cyanophage S-SZBM1 are likely to participate in auxiliary metabolism reactions after infection, such as photosynthesis (*psbA*, *cepT*, *petF*, *speD*, *hli*), carbon metabolism (*talC*, *cp12*), nucleotide synthesis and metabolism (carbamoyltransferase), cell protection (*hsp*, *prnA*, *prdx*, *nrdC*), and other cell metabolism (*phoH*, *mazG*, 2OG-Fe(II) oxygenase, *cobS*, cytidylyltransferase). All of these AMGs are conserved in cyanophage S-SZBM1 genome, with the exception of the gene *hli*, while several of them (*psbA*, *mazG*, *phoH*, *hsp*, *hli*, and *cobS*) are described as T4-like core genes.

Phage-encoded photosynthesis-related genes are the most representative AMGs to investigate phage–host interactions. Generally, utilization of cyanophage-encoded photosynthesis genes can boost the host photosynthetic performance during infection and, therefore, meet the energy demands for viral replication [48,51,52]. The *psbA* and *psbD* genes for the core photosystem II reaction center D1 and D2 encoded by genomes of phages were most well studied; these two proteins might potentially replenish the rapidly damaged host homologous proteins during infection [53,54]. More recent studies showed that cyanophage *psbA* under high light conditions would be up-regulated, which may increase the rate of delivery of D1 protein to PSII, resulting in an increase in the rate of photophosphorylation and more energy for cyanophage development [7,55]. Among cyanophage S-SZBM1 and its most related 32 cyanophages, the *psbA* gene is present in all cyanophages (100%), while the distribution of *psbD* gene is more sporadic (72.73%), and this finding is consistent with previous studies [18,19,43,51,56,57]. In addition, the *speD* gene found in cyanophage S-SZBM1, which is a key enzyme in the biosynthesis of the polyamines spermine and spermidine, might involve in regulating the host PSII reaction center during infection [58]. The *speD* gene was also reported to benefit phages in that polyamines are charge balance contributors in T4-like phage genome packaging processes [52,59]. Moreover, cyanophage S-SZBM1 has a *hli* gene, which has been reported to safeguard the host’s photosynthetic apparatus by dissipating excess light energy [13,60]. Cyanophage S-SZBM1 also contains a photosynthetic electron transport gene *petF*, and gene *cpeT* which might be important for light-harvesting phycobiliproteins assembly during infection [61]. As has been found in many other cyanophages, cyanophage S-SZBM1 contains a set of photosynthetic related genes potentially functioning in the alteration of host metabolic process during infection.

In addition to photosystems, cyanophages also influence phototrophic energy by regulating *zwf*, *gnd*, and *talc* genes encoding pentose phosphate pathways (PPP) enzymes, as well as *cp12*, a gene encoding an allosteric repressor of two Calvin cycle enzymes [48,49]. Cyanophage S-SZBM1 was found to carry both *cp12* and *talC.* The gene *cp12* directs carbon flux from the Calvin cycle to the PPP, while *talc* encodes a transaldolase. The synergetic expression of *cp12* and *talC* has been reported to increase the host NADPH/NADP ratio upon infection, while phage-augmented NADPH production subsequently fuels deoxynucleotide biosynthesis for phage replication [17].

Cyanophage S-SZBM1 carries a carbamoyltransferase homology, which can catalyze the first step of de novo pyrimidine biosynthesis and subsequently increase nucleotide synthesis during infection and, therefore, facilitate the viral genome replication [62]. In addition to benefiting phage nucleotide synthesis, some cyanophage-encoded genes, such as cell protection genes, can control host environment-sensing regulatory proteins to ensure viral replication under unfavorable conditions. A small heat shock protein, which is characterized by an α-crystallin domain surrounded by highly variable N-terminal and C-terminal regions and plays a role in proteostasis at high temperature, is also present in the genome of cyanophage S-SZBM1 [63]. As well, a gene *prnA* carried by cyanophage S-SZBM1, which can catalyze the first step in the biosynthesis of pyrrolnitrin, was speculated to provide antibiotic protection to its host [14,64,65,66]. Moreover, cyanophage S-SZBM1 also possesses *prdx* and *nrdC* genes, which might be involved in cell protection. For instance, they were suggested to enhance the defense against potential oxidative damage and manipulate stress responses in host cells via changes in redox state, respectively [67,68].

Cyanophage S-SZBM1 possesses a gene, *phoH*, which has the highest frequency of occurrence in sequenced phage genomes among pho regulon [69]. As we know, the gene *pstS* has been proven to encode a phosphate-binding protein involved in phosphate transport, while *phoA* presumably catalyzes the removal of phosphate groups from a variety of substrates to free bound phosphates for viral use [68,70]. Similar to *pstS* and *phoA*, *phoH* has always been speculated to play a crucial role in assisting phage genome replication under phosphorus-depleted conditions [16,69]. However, no evidence has been provided for *phoH* to be related to phosphate stress, and the role of *phoH* in infected cells is still unclear.

The gene *mazG* encodes pyrophosphohydrolase, which is universally distributed in cyano-myoviruses [42,44,58]. Previous studies showed that the *mazG* gene in cyanophages is not clustered with the host *mazG* gene, suggesting cyanophage-encoded *mazG* might arise outside cyanobacteria [44,71]. Recent investigation has demonstrated that neither the *Synechococcus* nor cyanophage *mazG* demonstrated detectable hydrolytic activity towards (p)ppGpp [72]. Instead, the cyanophage *mazG* preferentially hydrolyze dGTP and dCTP deoxyribonucleotides from the host genomes, allowing it to reuse building blocks from its host to replicate the cyanophage genome [72].

Multiple copies of 2OG-Fe(II) oxygenase are widely present in cyanophage S-SZBM1 (nine copies) as well as other cyano-myoviruses, while all of these 2OG-Fe(II) oxygenases are conserved in cyanophage S-SZBM1 genome. According to previous studies, 2OG-Fe(II) oxygenases encoded in phages were proposed to be functioning in DNA repair, because they are always located adjacent to DNA repair genes, such as *uvsX*, *uvsY*, and *uvsW* [44,73]. Unlike early findings, 2OG-Fe(II) oxygenases in cyanophage S-SZBM1 are scattered throughout the genome and are not present between *uvsY* and *uvsW*. Previous study showed that the 2OG-Fe(II) oxygenase genes phylogenetically divided into several groups, even where the genes came from the same genome, indicating gene duplication followed by divergence or multiple origins [74].

A cobalt chelatase gene *cobS*, which was predicted to catalyze the final step of vitamin B_12_ biosynthesis, is also possessed by cyanophage S-SZBM1 [75]. According to the function of bacterial *cobS*, phage-encoded *cobS* was presumed to increase the activity of ribonucleotide reductases for DNA synthesis [76]. However, the cyanophage *cobS*, including the one found in S-SZBM1 genome, is phylogenetically distinct from the host counterpart, suggesting that it might not originate from its host and probably has different functional properties [77].

Finally, we also identified a gene encoding cytidylyltransferase-like protein, which was found in various phages, such as S-WAM1, P-TIM40, P-RSM6, etc. [18,78]. The homologous genes have been reported to encode glycerol-3-phosphate cytidylyltransferase, which may be involved in biosynthesis of lipoteichoic acid and is required for bacterial cell wall biogenesis [79,80,81]. However, the function of cyanophage S-SZBM1-encoded cytidylyltransferase remains unclear.

### 3.4. Cyanophages Structural Proteome

The LC-MS based proteomics analysis has led to the identification of 30 proteins from cyanophage S-SZBM1 (Mascot score > 50), including 24 structural proteins (a total of 32 structural proteins were detected in cyanophage S-SZBM1 genome), one enzyme (cytidylyltransferase), and five proteins with unknown function (Table 1). As a T4-like phage, cyanophage S-SZBM1 assembly consists of three independent stages, including head (proteins of S-SZBM1_72, S-SZBM1_176-178, S-SZBM1_180, S-SZBM1_185, S-SZBM1_196, S-SZBM1_198, and S-SZBM1_199 were detected), tail (proteins of S-SZBM1_61, S-SZBM1_69, S-SZBM1_74, S-SZBM1_174, S-SZBM1_181, S-SZBM1_182, S-SZBM1_197, S-SZBM1_204, S-SZBM1_206, S-SZBM1_209, and S-SZBM1_210 were detected), and long tail fiber (no protein was detected) assembly pathways [82].

Portal vertex protein (S-SZBM1_180) is involved in the formation of a membrane-spanning initiation complex; scaffolding proteins (S-SZBM1_177, S-SZBM1_178) are responsible for the formation of a scaffolding core of prohead on this initiation complex [83]. Around this scaffolding core, the major capsid protein (S-SZBM1_176) builds an outer shell [84,85]. Then, enzyme zymogen (S-SZBM1_178) activates itself to be a T4 PPase and cleaves related proteins to free the space for DNA genome [86,87,88]. When the genome is loaded into the head, terminase will initiate the DNA packing, which consists of multiple copies of small subunit (S-SZBM1_196) and large subunit (S-SZBM1_185). Head completion protein (S-SZBM1_72) and neck protein (S-SZBM1_198, S-SZBM1_199) promote maturation of the head, bind with portal vertex protein, and set up a site for the attachment of phage tail [88,89]. The tail assembly is divided into several modules. A wedge is formed by multicopy baseplate wedge subunit protein (S-SZBM1_74, S-SZBM1_204, S-SZBM1_206, S-SZBM1_209, S-SZBM1_210); the central hub consists of hub subunit protein and tail lysozyme (S-SZBM1_61, S-SZBM1_69); then, six identical wedges surround a central hub to make up a baseplate [82]; multicopy tube protein (S-SZBM1_181) and sheath protein (S-SZBM1_182) separately build a tail tube and its outer retractable sheath [90]; this stage of assembly is completed by tail completion protein (S-SZBM1_174) and sheath stabilizer (S-SZBM1_197) [82]. According to the result of TEM images, long tail fiber is absent in S-SZBM1, which explains the missing of corresponding genes or translated products from its genome and proteome. The lack of long tail fibers or related gene/protein has also been reported in several other closely related cyanophages, such as cyanophages S-WAM1, P-RSM6, and P-TIM40, which differs from typical T4-like phages.

The LC-MS analysis also revealed two proteins, namely YadA domain-containing structural protein and DUF680 domain-containing protein, that have been commonly found in a number of cyanophages but not in classic T4-like assembly. They were thought of as the targets of positive selection to adapt sub-optimal hosts and to promote genetic diversity of phage populations [91]. The *YadA* gene was first identified in a Gram-negative bacillus-shaped bacterium *Yersinia enterocolitica*, and was reported to encode an agglutinating adhesin (YadA, *Yersinia* adhesion), a homotrimeric outer membrane protein belonging to the trimeric autotransporter adhesin (TAA) family [92]. This protein forms a fibrillar matrix at bacterial surface and acts as an adhesin and an invasin, mediating *Yersinia* adherence and enhancing its entry to epithelial cells [93,94]. In phages, its homolog, *YadA* domain-containing structural protein, could be found in various phages, including *Enterobacteria* phage ΦEco32, *Pectobacterium* phage My1, *Synechococcus* phages Syn19 and Syn30, and *Prochlorococcus* phages P-HM1 and P-HM2 [95]. A previous study suggested that *YadA* domain-containing structural protein encoded by *Enterobacteria* phage T7 takes part in assembly of phage tail fiber, and its membrane adhesion-like domain may play a role in the infection attachment to the host cell [96].

DUF680 domain-containing protein (PhCOG173) is a conserved cyanophage-specific protein. The gene encoding this protein is reported to neighbors and cooperates with multiple genes that are shared by phage–host to achieve various functions. Previous studies revealed that *phoH* gene was identified as a phosphate regulon in *Escherichia coli*. This gene was defined as a signature core gene in cyanomyovirus, which was related to pho boxes that could be found upstream of DUF680 in some reports, but the roles of these genes in cyanobacteria are still unclear [16,68,97]. *NrdC* locates next to DUF680 in S-SZBM1 genome. This gene is known to be a core gene that participates in regulation of cellular redox state, which may work with DUF680 to impact host metabolic processes toward nucleotide production [17,98]. More recent studies indicated that DUF680 protein has various mutations in cyanophage infecting the oceanic cyanobacterium *Synechococcus* [68,91].

## 4. Conclusions

In this study, a novel cyanophage, S-SZBM1, infecting *Synechococcus* CB0101 was isolated and characterized based on its physiological characteristics, taxonomy, and genomic and proteomic features. As a myovirus, cyanophage S-SZBM1 does not have a broad host range as expected and only shows lysis ability to its host strain. Surprisingly, up to 25 AMGs are encoded by cyanophage S-SZBM1, which potentially re-program the phage–host interactions. These AMGs are mainly involved in photosynthesis, carbon metabolism, nucleotide synthesis and metabolism, cell protection, and other cell metabolism. Taxonomy analysis showed that cyanophage S-SZBM1 belongs to a new genus under the family *Kyanoviridae*. According to the assembly pathways in bacteriophage T4, the structural assembly processes during infection were also speculated based on the identified structural genes of cyanophage S-SZBM1. Taken together, this study represents the structural and genomic characterization of a newly isolated and distinct cyanophage.

## Figures and Tables

**Table 1:** Confirmed proteins encoded by genes in cyanophage S-SZBM1 (score > 50).

Locus Tag Number	Putative Function	Mass (kDa)	aa Length	Peptides	Coverage	Score	*p*-Value	emPAI
S-SZBM1_176	Major capsid protein	48.46	452	27	42.3%	50,627	0	27.64
S-SZBM1_180	Portal vertex of the head	62.08	535	19	32.5%	4979	0	2.83
S-SZBM1_195	Hypothetical protein	9.06	87	2	43.7%	953	5.01 × 10^−96^	2.66
S-SZBM1_174	Tail completion protein	21.59	191	5	22.0%	491	7.94 × 10^−50^	1.38
S-SZBM1_204	Baseplate wedge	55.49	511	13	31.3%	6096	0	1.37
S-SZBM1_54	YadA domain-containing structural protein	47.55	444	12	35.1%	2493	5.01 × 10^−250^	1.24
S-SZBM1_53	Structural protein	76.76	734	13	27.7%	4393	0	1.21
S-SZBM1_90	DUF680 domain-containing protein	12.58	122	2	25.4%	1159	1.26 × 10^−116^	1.06
S-SZBM1_203	Baseplate wedge tail fiber connector	269.22	2446	53	27.1%	17,271	0	1.03
S-SZBM1_206	Baseplate wedge protein	51.38	498	9	24.9%	2195	3.16 × 10^−220^	0.98
S-SZBM1_136	Virion structural protein	21.23	199	2	24.1%	7040	0	0.80
S-SZBM1_74	Baseplate wedge component	33.50	292	6	19.9%	858	1.58 × 10^−86^	0.76
S-SZBM1_182	Tail sheath protein	100.56	924	12	17.9%	4763	0	0.72
S-SZBM1_197	Tail sheath stabilizer and completion protein	30.07	265	4	22.3%	1414	3.98 × 10^−142^	0.69
S-SZBM1_200	Structural protein	215.00	2073	25	17.0%	10,556	0	0.67
S-SZBM1_199	Neck protein	31.07	264	5	14.0%	541	7.94 × 10^−55^	0.66
S-SZBM1_52	Structural protein	129.96	1192	16	20.2%	5256	0	0.60
S-SZBM1_208	Baseplate wedge initiator	724.20	6619	71	13.5%	11,794	0	0.41
S-SZBM1_139	Hypothetical protein	48.43	457	4	10.5%	2382	6.31 × 10^−239^	0.39
S-SZBM1_205	Baseplate wedge initiator	65.69	615	6	10.9%	1559	1.26 × 10^−156^	0.34
S-SZBM1_178	Prohead core scaffolding protein and protease	23.91	215	2	21.9%	361	7.94 × 10^−37^	0.30
S-SZBM1_140	Cytidylyltransferase	40.96	362	2	13.5%	169	1.26 × 10^−17^	0.26
S-SZBM1_209	Baseplate wedge subunit	76.50	691	5	9.7%	507	2.00 × 10^−51^	0.23
S-SZBM1_190	Virion structural protein	46.62	424	3	7.1%	461	7.94 × 10^−47^	0.23
S-SZBM1_177	Prohead core protein	37.27	339	2	19.8%	248	1.58 × 10^−25^	0.19
S-SZBM1_65	Hypothetical protein	60.80	536	3	10.8%	183	5.01 × 10^−19^	0.17
S-SZBM1_181	Tail tube protein	24.75	224	1	7.6%	98	1.58 × 10^−10^	0.14
S-SZBM1_207	Structural protein	58.61	532	1	2.6%	245	3.16 × 10^−25^	0.12
S-SZBM1_51	Hypothetical protein	30.51	287	1	4.9%	120	1.00 × 10^−12^	0.11
S-SZBM1_198	Neck protein	32.36	291	1	8.6%	74	3.98 × 10^−8^	0.10

## Data Availability

The complete genome sequence of cyanophage S-SZBM1 was submitted to the GenBank database under accession number OL473597.

## Supplementary Materials

The following supporting information can be downloaded at: https://www.mdpi.com/article/10.3390/v14050887/s1, Table S1: Genome annotations of cyanophage S-SZBM1; Table S2: T4-like core genes in cyanophage S-SZBM1 genome; Table S3: tRNA genes predicted in the genome of cyanophage S-SZBM1; Table S4: Homologs of cyanophage S-SZBM1 predicted in the non-redundant database based on the complete nucleotide sequence.
