# Supplementary material for: Isolation and Characterization of a Novel Cyanophage Encoding Multiple Auxiliary Metabolic Genes
Source: Viruses. 2022 Apr 24;14(5):887. doi: 10.3390/v14050887 (PMC9146016; doi:10.3390/v14050887)
Supplement: Supplementary file 1 [file viruses-14-00887-s001.zip › viruses-1644151-supplementary.pdf]

viruses

MDPI

Supplementary Materials

Isolation and Characterization of a Novel Cyanophage Encoding Multiple Auxiliary Metabolic Genes

Table S1. Genome annotations of cyanophage S-SZBM1.

Gene No.	Start	End	aa length	Strand	Related phage or microbes	Query cover	*e*-value (≤10⁻⁵)	Percent identity	Accession	Conserved domain Accession	Conserved domain *e*-value (≤10⁻⁵)	Putative function
1	69	269	66	-	Cyanophage P-RSM6	86%	2.00E-20	73.68%	YP_007675177.1			Hypothetical protein
2	266	433	55	-	Synechococcus phage S-WAM1	100%	8.00E-26	80.00%	YP_009325064.1			Hypothetical protein
3	426	557	43	-	n/a	n/a	n/a	n/a	n/a			Hypothetical protein
4	1028	1645	205	-	Synechococcus phage S-WAM1	99%	5.00E-108	71.43%	YP_009325061.1			Hypothetical protein
5	1653	2084	143	-	Synechococcus phage S-RIM8 A.HR1	97%	4.00E-42	52.11%	YP_007518150.1			Hypothetical protein
6	2087	2251	54	-	n/a	n/a	n/a	n/a	n/a			Hypothetical protein
7	2254	2469	71	-	Synechococcus phage S-WAM1	100%	6.00E-30	77.46%	YP_009325054.1			Hypothetical protein
8	2492	2800	102	-	Synechococcus phage syn9	95%	3.00E-16	44.00%	YP_717757.1			Hypothetical protein
9	2942	3118	58	-	n/a	n/a	n/a	n/a	n/a			Hypothetical protein
10	3120	3281	53	-	Synechococcus phage S-RIM8 A.HR1	100%	9.00E-27	88.68%	YP_007518154.1			Hypothetical protein
11	3295	3561	88	-	n/a	n/a	n/a	n/a	n/a			Hypothetical protein
12	3585	3773	62	-	Synechococcus phage S-RIM8 A.HR1	100%	2.00E-23	66.13%	YP_007518141.1			Hypothetical protein
13	3851	4126	91	-	Alphaproteobacteria bacterium	80%	1.00E-04	33.75%	NDB82294.1			Hypothetical protein

14	4192	4383	63	-	Synechococcus phage syn9	79%	6.00E-17	66.00%	YP_717747.1			Hypothetical protein
15	4383	4646	87	-	Synechococcus phage S-RIM8	98%	1.00E-39	73.26%	YP_009782974.1			Hypothetical protein
16	4726	4911	61	-	n/a	n/a	n/a	n/a	n/a			Hypothetical protein
17	4988	5416	142	-	Bacterium	99%	2.00E-62	70.42%	NBX51468.1			Hypothetical protein
18	5598	5780	60	-	n/a	n/a	n/a	n/a	n/a			Hypothetical protein
19	5773	5943	56	-	n/a	n/a	n/a	n/a	n/a			Hypothetical protein
20	5943	6212	89	-	Uncultured Mediterranean phage uvMED	71%	2.00E-15	56.92%	BAR40008.1			Hypothetical protein
21	6184	6330	48	-	n/a	n/a	n/a	n/a	n/a			Hypothetical protein
22	6419	6583	54	-	Synechococcus phage S-CBM2	90%	1.00E-10	53.06%	AFK66434.1			Hypothetical protein
23	6583	6831	82	-	Synechococcus phage S-CBM2	100%	2.00E-49	91.46%	AFK66448.1			Hypothetical protein
24	7405	8574	389	-	Cyanophage P-RSM6	98%	9.00E-177	63.61%	YP_007675203.1			Hypothetical protein
25	8655	8915	86	-	Cyanophage P-RSM6	70%	4.00E-09	42.62%	YP_007675204.1			Hypothetical protein
26	8912	9106	64	-	Synechococcus phage S-WAM1	98%	6.00E-23	61.90%	YP_009325044.1			Hypothetical protein
27	9116	9244	42	-	Bacterium	100%	6.00E-11	64.29%	NDG31949.1			Hypothetical protein
28	9241	9327	28	-	n/a	n/a	n/a	n/a	n/a			Hypothetical protein
29	9327	9629	100	-	Bacterium	100%	1.00E-57	84.00%	NBX50893.1	cd22268	2.05E-44	Rare lipoprotein A, RlpA
30	9666	10202	178	-	Cyanophage P-RSM6	90%	1.00E-74	65.64%	YP_007675205.1			Hypothetical protein
31	10220	10897	225	-	Euryarchaeota archaeon	98%	2.00E-133	77.83%	MBM4242076.1	pfam13529	9.62E-13	C39 family peptidase
32	10912	11418	168	-	Synechococcus phage S-WAM1	98%	2.00E-66	60.24%	YP_009325040.1	pfam13759	1.58E-10	Putative 2OG-Fe(II) oxygenase
33	11512	12756	414	-	Synechococcus phage S-WAM1	100%	0.00E+00	80.43%	YP_009325039.1	COG1061	1.32E-25	Helicase
34	12840	13163	107	-	Chitinophagia bacterium	98%	5.00E-06	27.52%	NDF98420.1			Hypothetical protein
35	13182	13493	103	-	n/a	n/a	n/a	n/a	n/a			Hypothetical protein
36	13486	13596	36	-	n/a	n/a	n/a	n/a	n/a			Hypothetical protein

37	13598	13759	53	-	Synechococcus phage S-H25	94%	2.00E-12	64.00%	QIN97213.1			Hypothetical protein
38	13756	14088	110	-	Synechococcus phage S-WAM1	100%	3.00E-41	59.09%	YP_009325030.1			Hypothetical protein
39	14085	14219	44	-	Deltaproteobacteria bacterium	100%	2.00E-07	50.00%	MBT89952.1			Hypothetical protein
40	14233	14403	56	-	Flavobacteriales bacterium TMED113	94%	7.00E-10	50.94%	OUV52660.1			Hypothetical protein
41	14428	14793	121	-	Deltaproteobacteria bacterium	99%	2.00E-47	65.83%	MBT89954.1			Hypothetical protein
42	14939	15643	234	-	Synechococcus phage S-WAM1	100%	8.00E-153	85.47%	YP_009325027.1	pfam08007	1.17E-12	Cupin superfamily protein
43	15640	16311	223	-	Synechococcus phage S-WAM1	83%	2.00E-82	63.54%	YP_009325026.1	pfam13759	3.69E-12	Putative 2OG-Fe(II) oxygenase
44	16292	16975	227	-	Cyanophage P-RSM6	98%	7.00E-127	76.34%	YP_007675218.1	pfam13759	1.05E-29	Putative 2OG-Fe(II) oxygenase
45	16975	17445	156	-	Synechococcus phage S-WAM1	85%	5.00E-62	67.16%	YP_009325024.1			Hypothetical protein
46	17448	19034	528	-	Synechococcus phage S-WAM1	100%	0.00E+00	77.65%	YP_009325023.1	pfam04820	7.56E-97	Tryptophan halogenase, PrnA
47	19041	19589	182	-	Deltaproteobacteria bacterium	91%	4.00E-72	63.69%	MBT89960.1			Hypothetical protein
48	19596	20396	266	-	Rhodobacteraceae bacterium	100%	4.00E-145	86.09%	MBT25064.1			Hypothetical protein
49	20429	21232	267	-	Deltaproteobacteria bacterium	100%	2.00E-150	86.14%	MBT89962.1			Hypothetical protein
50	21281	22156	291	-	Myoviridae sp.	94%	4.00E-30	44.83%	QMP83641.1			Hypothetical protein
51	22166	23029	287	-	Cyanophage P-RSM6	98%	2.00E-136	71.83%	YP_007675227.1			Hypothetical protein
52	23049	26627	1192	-	Cyanophage P-RSM6	100%	0.00E+00	90.60%	YP_007675228.1			Structural protein
53	26672	28876	734	-	Synechococcus phage S-WAM1	100%	0.00E+00	65.31%	YP_009325015.1			Structural protein
54	28884	30218	444	-	Synechococcus phage S-WAM1	100%	0.00E+00	75.90%	YP_009325014.1			YadA domain-containing structural protein
55	30251	30475	74	-	Synechococcus phage S-CBM2	89%	6.00E-17	54.55%	AFK66352.1			Hypothetical protein
56	30492	30635	47	-	Alphaproteobacteria bacterium	87%	2.00E-16	85.37%	NDB86287.1			Hypothetical protein
57	30694	31194	166	-	Synechococcus phage S-E7	100%	4.00E-28	38.95%	AYR02149.1			DNA endonuclease V
58	31930	32121	63	-	Synechococcus phage S-WAM1	100%	8.00E-24	66.67%	YP_009325011.1			Hypothetical protein

59	32257	32541	94	-	Cyanophage P-RSM6	97%	5.00E-28	53.76%	YP_007675234.1			Hypothetical protein
60	32546	32734	62	-	Cyanophage P-RSM1	100%	2.00E-14	50.00%	YP_007877620.1			Hypothetical protein
61	32740	35295	851	-	Cyanophage P-RSM6	99%	0	59.91%	YP_007675236.1	PHA02596	3.30E-11	Baseplate hub subunit and tail lysozyme
62	35295	35783	162	-	Cyanophage P-RSM6	97%	3.00E-72	63.29%	YP_007675237.1	cd16338	6.32E-11	CpeT-like protein, CpeT
63	35767	36372	201	-	Synechococcus phage S-WAM1	68%	3.00E-70	71.01%	YP_009325005.1	pfam13640	2.09E-11	2OG-Fe(II) oxygenase
64	36436	36648	70	-	Synechococcus phage S-H25	100%	7.00E-24	62.86%	QIN97220.1	smart01093	1.07E-12	CP12 carbon metabolic regulator, CP12
65	36673	38283	536	-	Deltaproteobacteria bacterium	98%	0.00E+00	52.82%	MBT89976.1			Hypothetical protein
66	38283	40625	780	-	Cyanophage P-RSM6	99%	0.00E+00	40.92%	YP_007675241.1	cd00736	2.27E-06	Lambda lysozyme
67	40630	43311	893	-	Cyanophage P-RSM6	63%	7.00E-80	34.05%	YP_007675242.1			Hypothetical protein
68	43311	43502	63	-	Cyanophage P-RSM6	100%	4.00E-21	65.08%	YP_007675243.1	PHA02078	2.06E-13	Baseplate hub assembly catalyst
69	43508	44209	233	-	Rhodobacteraceae bacterium	100%	2.00E-133	78.11%	MBT25751.1	pfam12322	3.77E-14	Baseplate protein
70	44235	44984	249	-	Synechococcus phage S-WAM1	85%	1.00E-74	53.27%	YP_009324999.1			Hypothetical protein
71	44981	45451	156	-	Synechococcus phage ACG-2014i	98%	2.00E-13	26.52%	YP_009140793.1	PHA02577	1.75E-08	DNA end protector protein
72	45448	45882	144	-	Synechococcus phage S-WAM1	100%	2.00E-89	84.03%	YP_009324997.1	PHA02552	1.75E-73	Head completion protein
73	45881	46894	337	+	Synechococcus phage S-WAM1	94%	2.00E-123	54.63%	YP_009324996.1			Baseplate tail tube cap
74	46894	47772	292	+	Cyanophage P-RSM6	96%	3.00E-117	55.67%	YP_007675249.1	PHA02578	4.04E-14	Baseplate wedge component
75	48079	49020	313	-	Synechococcus phage S-WAM1	100%	0.00E+00	82.43%	YP_009324991.1	PHA02550	3.69E-126	Single stranded DNA-binding protein
76	49125	49487	120	-	Synechococcus phage S-WAM1	97%	6.00E-56	72.88%	YP_009325210.1	pfam08855	2.31E-07	DUF1825 domain-containing protein
77	49484	49729	81	-	Cyanophage P-TIM40	97%	4.00E-33	70.89%	YP_009188078.1	pfam16805	3.00E-20	Late promoter transcription accessory protein
78	49726	50370	214	-	Nonlabens xiamenensis	97%	1.00E-85	62.68%	WP_206052197.1	pfam12705	4.49E-08	PD-(D/E)XK nuclease
79	50409	51161	250	-	Synechococcus phage S-WAM1	100%	3.00E-168	88.89%	YP_009325206.1	pfam02562	9.38E-36	PhoH
80	51163	51393	76	-	Synechococcus phage S-WAM1	100%	6.00E-37	80.26%	YP_009325205.1			Hypothetical protein

81	51395	51574	59	-	Synechococcus phage S-SCSM1	100%	2.00E-32	94.92%	QFG06454.1			Hypothetical protein
82	51574	52257	227	-	Synechococcus phage S-CBM2	100%	9.00E-145	83.05%	AFK66378.1	PRK00847	1.13E-81	Thymidylate synthase
83	52254	52508	84	-	Cyanophage P-RSM6	67%	1.00E-14	57.63%	YP_007675039.1			Hypothetical protein
84	52505	52774	89	-	bacterium	100%	6.00E-16	46.67%	NBV27774.1			Hypothetical protein
85	52775	53206	143	-	Synechococcus phage S-WAM1	100%	7.00E-79	79.02%	YP_009325200.1			Hypothetical protein
86	53208	54086	292	-	Synechococcus phage S-WAM1	100%	3.00E-159	75.00%	YP_009325199.1	PHA02567	1.38E-106	Ribonuclease H
87	54092	54331	79	-	Euryarchaeota archaeon	97%	1.00E-30	70.51%	MBG46620.1			Hypothetical protein
88	54363	54734	123	-	Synechococcus phage S-WAM1	100%	7.00E-65	71.54%	YP_009325197.1			Hypothetical protein
89	54734	54985	83	-	Synechococcus phage S-WAM1	100%	6.00E-43	78.31%	YP_009325196.1	cd03029	7.98E-16	Glutaredoxin, NrdC
90	55050	55418	122	-	Synechococcus phage S-SCSM1	100%	4.00E-16	39.84%	QFG06402.1			DUF680 domain-containing protein
91	55564	55878	104	-	Cyanophage P-RSM6	96%	2.00E-34	63.73%	YP_007675047.1			Hypothetical protein
92	55878	59174	1098	-	Cyanophage P-RSM6	98%	0.00E+00	45.44%	YP_007675048.1			Hypothetical protein
93	59236	59361	41	-	n/a	n/a	n/a	n/a	n/a			Hypothetical protein
94	59358	59897	179	-	Synechococcus phage S-PM2	93%	6.00E-61	54.14%	YP_195042.2			Hypothetical protein
95	59894	60076	60	-	Synechococcus phage S-PM2	100%	2.00E-32	88.33%	CFW42160.1			Hypothetical protein
96	60069	60359	96	-	Synechococcus phage S-H9-2	50%	5.00E-18	77.08%	QPB08477.1			Hypothetical protein
97	60356	60541	61	-	bacterium	100%	2.00E-17	57.38%	NDG30122.1			Hypothetical protein
98	60544	60666	40	-	n/a	n/a	n/a	n/a	n/a			Hypothetical protein
99	60663	60869	68	-	Synechococcus phage S-PM2	97%	1.00E-34	83.33%	YP_195055.1			Hypothetical protein
100	60866	61051	61	-	Synechococcus phage S-PM2	100%	4.00E-35	95.08%	YP_195053.1			Hypothetical protein
101	61048	61209	53	-	Synechococcus phage S-PM2	100%	7.00E-29	94.34%	YP_195052.1			Hypothetical protein
102	61282	61662	126	-	Synechococcus phage S-PM2	100%	1.00E-71	81.75%	YP_195048.1	COG4453	4.91E-07	DUF1778 domain-containing protein

103	61746	62138	130	-	Alphaproteobacteria bacterium	93%	2.00E-65	77.87%	NDB85592.1			Hypothetical protein
104	62135	62542	135	-	Cylindrospermopsis curvispora	89%	2.00E-58	73.44%	WP_187707554.1			Membrane protease subunit
105	62539	62661	40	-	Synechococcus phage S-PM2	100%	4.00E-17	82.50%	YP_195045.1			Hypothetical protein
106	62661	62966	101	-	Synechococcus phage S-PM2	94%	4.00E-47	74.74%	YP_195044.1			Hypothetical protein
107	62953	63180	75	-	Synechococcus phage S-CBS4	81%	1.00E-20	59.02%	YP_005098305.1			Rnf-Nqr, nitrogen fixation protein-like protein
108	63253	64332	359	-	Synechococcus phage S-PM2	100%	0.00E+00	93.04%	YP_195211.1	TIGR01151	0.00E+00	Photosystem II D1 protein, PsbA
109	64462	64737	91	-	n/a	n/a	n/a	n/a	n/a			Hypothetical protein
110	64727	64900	57	-	Synechococcus phage S-WAM1	89%	2.00E-09	49.02%	YP_009325186.1			Hypothetical protein
111	65166	65690	174	-	Synechococcus phage S-H9-1	98%	3.00E-91	73.14%	QPB08144.1	COG0678	1.16E-81	Peroxiredoxin, Prdx
112	65674	65919	81	-	Synechococcus phage S-SRM01	83%	3.00E-24	66.18%	QPX48290.1			Hypothetical protein
113	65916	66206	96	-	Synechococcus phage S-CBM2	97%	2.00E-55	88.30%	AFK66341.1	CHL00134	2.73E-49	Ferredoxin I, PetF
114	66292	66486	64	-	Alphaproteobacteria bacterium	100%	3.00E-32	82.81%	NDB87304.1			High light inducible protein, Hli
115	66537	66806	89	-	Uncultured Mediterranean phage uvMED	84%	2.00E-11	41.33%	BAR31741.1			Hypothetical protein
116	67001	67237	78	-	Caulobacteraceae bacterium	92%	9.00E-39	83.33%	NBW15424.1			Hypothetical protein
117	67270	67890	206	-	Synechococcus phage S-WAM1	100%	3.00E-136	87.86%	YP_009325178.1	pfam13640	4.23E-08	2OG-Fe(II) oxygenase superfamily
118	67887	68096	69	-	Synechococcus phage S-WAM1	100%	2.00E-35	81.16%	YP_009325177.1	PHA02360	3.86E-14	Hypothetical protein
119	68301	68819	172	-	Synechococcus phage S-WAM1	100%	4.00E-87	71.51%	YP_009325176.1	pfam13640	1.59E-15	2OG-Fe(II) oxygenase superfamily
120	68806	68955	49	-	n/a	n/a	n/a	n/a	n/a			Hypothetical protein
121	69001	69573	190	-	Cyanophage P-RSM6	98%	6.00E-94	68.98%	YP_007675057.1	pfam13759	3.92E-15	2OG-Fe(II) oxygenase superfamily
122	69545	69784	79	-	Euryarchaeota archaeon	89%	2.00E-41	91.55%	MBU37915.1			Hypothetical protein
123	69858	70079	73	-	Synechococcus phage S-WAM1	68%	2.00E-17	84.00%	YP_009325172.1			Hypothetical protein
124	70069	70236	55	-	Synechococcus phage S-WAM1	100%	4.00E-19	70.91%	YP_009325171.1			Hypothetical protein

125	70238	70891	217	-	Synechococcus phage Syn19	99%	3.00E-102	65.28%	YP_004324023.1	PRK01362	2.69E-94	Transaldolase-like protein, fructose-6-phosphate aldolase, TalC
126	71049	71183	44	-	n/a	n/a	n/a	n/a	n/a			Hypothetical protein
127	71223	71381	52	-	Synechococcus phage S-H9-2	100%	8.00E-07	43.40%	QPB08418.1			Hypothetical protein
128	71534	71968	144	-	Synechococcus phage S-WAM1	97%	1.00E-29	48.23%	YP_009325167.1			Hypothetical protein
129	71931	73067	378	-	Synechococcus phage S-WAM1	100%	0.00E+00	83.07%	YP_009325166.1	PRK09101	0.00E+00	NrdB
130	73058	73225	55	-	Synechococcus phage S-RSM4	85%	5.00E-10	55.32%	YP_003097416.1			Hypothetical protein
131	73218	75527	769	-	Synechococcus phage S-WAM1	99%	0.00E+00	80.81%	YP_009325165.1	PHA02572	0.00E+00	NrdA
132	75524	76354	276	-	Cyanophage P-RSM6	100%	3.00E-154	72.83%	YP_007675068.1	PHA02540	2.13E-122	DNA primase
133	76503	76937	144	-	Synechococcus phage S-WAM1	98%	2.00E-50	57.34%	YP_009325163.1			Hypothetical protein
134	76939	77310	123	-	Synechococcus phage S-WAM1	98%	7.00E-51	68.03%	YP_009325162.1			Hypothetical protein
135	77307	77678	123	-	Euryarchaeota archaeon	100%	6.00E-64	77.24%	MBU38018.1	PHA02335	1.94E-46	Hypothetical protein
136	77694	78293	199	-	Synechococcus phage S-H35	97%	1.00E-68	60.30%	ARW56996.1			Virion structural protein
137	78380	78574	64	-	Synechococcus phage S-H35	100%	1.00E-30	82.81%	ARW56997.1	PHA02334	5.15E-24	Hypothetical protein
138	78608	78991	127	-	Synechococcus phage S-WAM1	99%	3.00E-30	60.94%	YP_009325157.1			Hypothetical protein
139	78988	80361	457	-	Synechococcus phage S-WAM1	57%	3.00E-60	64.81%	YP_009325156.1			Hypothetical protein
140	80389	81477	362	-	Synechococcus phage S-WAM1	100%	0.00E+00	76.99%	YP_009325155.1			Cytidylyltransferase
141	81477	82670	397	-	Synechococcus phage S-WAM1	100%	0.00E+00	69.77%	YP_009325154.1			Hypothetical protein
142	82670	84007	445	-	Synechococcus phage S-WAM1	100%	3.00E-86	34.41%	YP_009325153.1			Hypothetical protein
143	84035	84262	75	-	Hyphomonas sp. TMED31	100%	8.00E-27	64.00%	OUX85947.1			Hypothetical protein
144	84262	84912	216	-	Euryarchaeota archaeon	83%	2.00E-92	71.11%	MBJ29706.1	cd11541	2.00E-19	Nucleoside triphosphate pyrophosphohydrolase, MazG
145	84909	86291	460	-	Synechococcus phage S-WAM1	100%	0.00E+00	82.61%	YP_009325149.1	PHA02542	0.00E+00	DNA primase/helicase
146	86291	86854	187	-	Cyanophage P-RSM6	100%	3.00E-77	59.57%	YP_007675085.1	smart00702	2.70E-11	2OG-Fe(II) oxygenase superfamily

147	86844	87857	337	-	Euryarchaeota archaeon	99%	0.00E+00	85.42%	MBJ29703.1	COG0468	5.65E-24	Recombinase RecA
148	87854	90343	829	-	Synechococcus phage S-WAM1	95%	0.00E+00	83.60%	YP_009325146.1	PHA02528	0.00E+00	DNA polymerase
149	90343	90585	80	-	Cyanophage P-RSM6	96%	7.00E-29	64.94%	YP_007675088.1			Hypothetical protein
150	90563	90889	108	-	Euryarchaeota archaeon	94%	4.00E-27	51.96%	MBJ29700.1			Hypothetical protein
151	90861	91103	80	-	Cyanophage P-RSM6	98%	4.00E-33	68.35%	YP_007675090.1			Hypothetical protein
152	91167	91613	148	-	Synechococcus phage S-RIM2	99%	6.00E-95	87.76%	AOO00033.1	cd06470	1.80E-28	Heat shock protein, Hsp
153	91697	93136	479	-	Cyanophage P-RSM6	99%	0.00E+00	65.70%	YP_007675101.1	COG2192	2.46E-80	Carbamoyltransferase
154	93160	93576	138	-	Synechococcus phage S-WAM1	100%	1.00E-88	90.58%	YP_009325133.1	PHA02543	1.61E-74	Translation repressor protein
155	93573	93968	131	-	Cyanophage P-RSM6	96%	3.00E-62	69.05%	YP_007675103.1	PHA02593	2.99E-13	Clamp loader subunit
156	93952	94800	282	-	Synechococcus phage S-WAM1	100%	2.00E-158	74.22%	YP_009325131.1	COG0338	1.00E-38	Cytosine-specific DNA methyltransferase
157	94803	94943	46	-	Synechococcus phage S-CAM22	100%	2.00E-18	78.26%	YP_009321042.1			Hypothetical protein
158	94940	95503	187	-	Synechococcus phage S-WAM1	99%	6.00E-111	82.26%	YP_009325130.1	pfam13759	2.25E-13	Putative 2OG-Fe(II) oxygenase
159	95560	96504	314	-	Cyanophage P-RSM6	99%	0.00E+00	84.62%	YP_007675106.1	PHA02544	8.63E-166	Sliding clamp loader
160	96589	97326	245	-	Cyanophage P-RSM6	95%	6.00E-105	65.11%	YP_007675109.1	PHA02545	3.06E-51	Sliding clamp
161	97326	97628	100	-	Euryarchaeota archaeo	92%	5.00E-38	70.65%	MBU38050.1	pfam11753	8.31E-10	DUF3310 domain-containing protein
162	97687	98778	363	-	Synechococcus phage S-B43	97%	0.00E+00	75.07%	QDH50584.1	COG0714	4.41E-19	Putative cobalt chelatase subunit, CobS
163	98856	101012	718	-	Synechococcus phage S-WAM1	99%	0.00E+00	54.68%	YP_009325123.1			Peptidase
164	101181	101417	78	-	Prokaryotic dsDNA virus sp.	100%	2.00E-36	71.79%	QDP50089.1			Hypothetical protein
165	101530	101865	111	-	Synechococcus phage S-WAM1	100%	9.00E-53	69.37%	YP_009325122.1	PRK02770	4.18E-46	S-adenosylmethionine decarboxylase, SpeD
166	101867	103582	571	-	Cyanophage P-RSM6	99%	0.00E+00	69.37%	YP_007675115.1	PHA02562	0.00E+00	Recombination endonuclease subunit
167	103575	103835	86	-	Nonlabens xiamenensis	96%	2.00E-27	61.45%	WP_206052188.1			Hypothetical protein
168	103835	104863	342	-	Synechococcus phage S-WAM1	100%	0.00E+00	73.98%	YP_009325118.1	PHA02546	4.02E-133	Recombination-related endonuclease

169	104878	105099	73	-	Synechococcus phage S-B05]	100%	3.00E-16	47.95%	QCW22939.1			Hypothetical protein
170	105096	105575	159	-	Synechococcus phage S-WAM1	98%	9.00E-99	86.62%	YP_009325116.1	PHA02547	4.28E-64	RNA polymerase sigma factor for late transcription
171	105717	106160	147	-	Nonlabens xiamenensis	97%	2.00E-68	64.58%	YP_007675120.1	pfam16243	1.10E-23	Methylamine utilization protein
172	106153	107616	487	-	Synechococcus phage S-WAM1	100%	0.00E+00	85.83%	YP_009325114.1	PHA02558	0.00E+00	UvsW helicase
173	107616	108038	140	-	Synechococcus phage S-WAM1	100%	9.00E-86	85.71%	YP_009325113.1	pfam11056	5.61E-28	UvsY protein
174	108035	108610	191	-	Synechococcus phage S-WAM1	98%	1.00E-87	68.09%	YP_009325112.1	PHA02576	5.02E-14	Tail completion protein
175	108628	108762	44	-	Synechococcus phage S-CAM3	93%	9.00E-15	75.61%	YP_009321457.1			Hypothetical protein
176	109002	110360	452	-	Cyanophage P-RSM6	100%	0.00E+00	87.83%	YP_007675133.1	PHA02541	0.00E+00	Major capsid protein
177	110413	111432	339	-	Synechococcus phage S-WAM1	100%	2.00E-174	78.26%	YP_009325108.1	PHA02557	3.15E-64	Prohead core protein
178	111489	112136	215	-	Synechococcus phage S-WAM1	100%	2.00E-132	83.26%	YP_009325107.1	PHA00911	5.02E-94	Prohead core scaffolding protein and protease
179	112111	112356	81	-	Synechococcus phage S-WAM1	100%	4.00E-20	55.56%	YP_009325106.1			Hypothetical protein
180	112382	113989	535	-	Synechococcus phage S-WAM1	100%	0.00E+00	90.11%	YP_009325105.1	PHA02531	0.00E+00	Portal vertex of the head
181	114030	114704	224	-	Uncultured Mediterranean phage uvMED	94%	2.00E-90	61.99%	BAR35460.1	PHA02551	4.98E-63	Tail tube protein
182	114746	117520	924	-	Cyanophage P-RSM6	99%	0.00E+00	72.82%	YP_007675139.1	PHA02539	1.62E-178	Tail sheath protein
183	117610	117852	80	-	Synechococcus phage S-SRM01	98%	8.00E-44	86.08%	QPX48241.1			Hypothetical protein
184	117861	118082	73	-	uncultured Mediterranean phage uvMED	95%	1.00E-14	52.86%	BAR29000.1			T4-like Endonuclease VII
185	118082	119734	550	-	Synechococcus phage S-WAM1	99%	0.00E+00	88.95%	YP_009325102.1	PHA02533	0.00E+00	Terminase large subunit
186	119727	119942	71	-	Synechococcus phage S-H35	98%	7.00E-38	87.14%	ARW57058.1			Hypothetical protein
187	120369	120500	43	-	Synechococcus phage S-H9-1	100%	1.00E-15	76.74%	QPB08238.1			Hypothetical protein
188	120500	120736	78	-	Synechococcus phage S-RIM8	100%	8.00E-42	83.33%	AOO10471.1	PHA02325	5.53E-38	Hypothetical protein
189	120720	120944	74	-	Synechococcus phage S-WAM2	82%	1.00E-24	70.49%	YP_009324278.1			Hypothetical protein
190	121033	122307	424	-	Synechococcus phage S-RIM2	100%	0.00E+00	86.79%	AON98689.1			Virion structural protein

191	122311	122628	105	-	Synechococcus phage S-H25	96%	1.00E-36	66.02%	QIN97139.1			Hypothetical protein
192	122628	123125	165	-	Synechococcus phage ACG-2014f	49%	2.00E-40	88.89%	AIX16650.1			Hypothetical protein
193	123122	123259	45	-	Synechococcus phage S-WAM1	68%	4.00E-07	64.52%	YP_009325089.1			Hypothetical protein
194	123252	125375	707	-	Synechococcus phage S-WAM1	62%	4.00E-160	59.49%	YP_009325088.1			Hypothetical protein
195	125377	125640	87	-	Synechococcus phage S-H25	100%	1.00E-26	64.37%	QIN97141.1			Hypothetical protein
196	125684	126109	141	-	Synechococcus phage S-WAM1	100%	2.00E-76	82.98%	YP_009325086.1	pfam11053	8.80E-06	Terminase small subunit
197	126099	126896	265	-	Nonlabens xiamenensis	100%	4.00E-154	80.75%	WP_124981717.1	PHA02556	5.03E-95	Tail sheath stabilizer and completion protein
198	126896	127771	291	-	Synechococcus phage S-WAM1	100%	6.00E-173	80.07%	YP_009325084.1	PHA02555	7.46E-81	Neck protein
199	127773	128567	264	-	Nonlabens xiamenensis	100%	1.00E-158	78.20%	WP_124981715.1	PHA02554	6.07E-68	Neck protein
200	128574	134795	2073	-	Uncultured Mediterranean phage uvMED	99%	0.00E+00	67.88%	BAR31833.1			Structural protein
201	134884	135099	71	-	Synechococcus phage S-WAM1	81%	3.00E-23	77.59%	YP_009325081.1			Hypothetical protein
202	135099	135347	82	-	Uncultured Mediterranean phage uvMED	89%	7.00E-19	56.00%	BAR31831.1			Hypothetical protein
203	135347	142687	2446	-	Synechococcus phage S-WAM1	100%	0.00E+00	68.60%	YP_009325079.1	pfam16075	2.44E-26	Baseplate wedge tail fiber connector
204	142689	144224	511	-	Cyanophage P-RSM6	100%	0.00E+00	91.59%	YP_007675161.1	PHA02580	1.38E-09	Baseplate wedge
205	144259	146106	615	-	Synechococcus phage S-H38	90%	1.00E-160	48.06%	QPB07967.1			Baseplate wedge initiator
206	146110	147606	498	-	Synechococcus phage S-WAM1	100%	0.00E+00	70.48%	YP_009325076.1	PHA02582	2.14E-07	Baseplate wedge protein
207	147633	149231	532	-	Synechococcus phage S-WAM1	100%	0.00E+00	70.97%	YP_009325075.1	pfam14240	8.53E-38	Structural protein
208	149248	169107	6619	-	Synechococcus phage S-WAM1	100%	0.00E+00	72.64%	YP_009325074.1	pfam14240	1.62E-33	Baseplate wedge initiator
209	169115	171190	691	-	Cyanophage P-RSM6	100%	0.00E+00	78.08%	YP_007675166.1	PHA02553	1.63E-165	Baseplate wedge subunit
210	171192	171605	137	-	Synechococcus phage S-WAM1	85%	1.00E-50	65.81%	YP_009325072.1	PHA00415	4.92E-40	Baseplate wedge subunit
211	171605	171793	62	-	Synechococcus phage S-IOM18	98%	5.00E-26	82.26%	YP_008126388.1			Hypothetical protein
212	171906	172103	65	-	Synechococcus phage S-SRM01	80%	1.00E-16	63.46%	QPX48204.1			Hypothetical protein

213	172103	172252	49	-	Synechococcus phage S-WAM1	97%	8.00E-13	66.67%	YP_009325071.1	PHA02324	3.25E-06	Hypothetical protein
214	172521	172718	65	+	n/a	n/a	n/a	n/a	n/a			Hypothetical protein
215	172804	176607	1267	-	Cyanophage P-RSM6	98%	0.00E+00	51.26%	YP_007675170.1			Hypothetical protein
216	176607	176849	80	-	Synechococcus phage S-WAM1	96%	5.00E-35	76.62%	YP_009325069.1			Hypothetical protein
217	176910	177086	58	-	Synechococcus phage S-WAM1	91%	5.00E-13	56.60%	YP_009325067.1			Hypothetical protein
218	177109	177645	178	-	Rhodobacteraceae bacterium	89%	4.00E-59	57.14%	MBT26522.1			Hypothetical protein

Table S2. T4-like core genes in cyanophage S-SZBM1 genome. + means gene is in conserved domain and – means gene is not conserved.

Gene Description	Gene No. in S-SZBM1	The size of the gene (bp)	Conserved domain (*e*-value ≤ 10^{-5})
gp3 head-proximal tip of tail tube tail completion + sheath stabilizer protein	174	576	+
gp4 head completion protein	72	435	+
gp5 baseplate hub + tail lysozyme	61	2556	+
gp6 baseplate wedge	209	2076	+
gp8 baseplate wedge	204	1536	+
gp13 neck protein	199	795	+
gp14 neck protein	198	876	+
gp15 proximal tail sheath stabilization	197	798	+
gp16 terminase DNA packaging enzyme,small subunit	196	426	+
gp17 terminase DNA packaging enzyme,large subunit	185	1653	+
gp18 tail sheath monomer	182	2775	+
gp19 tail tube monomer	181	675	+
gp20 portal vertex protein of head	180	1608	+
gp21 prohead core scaffold and protease	178	648	+
gp22 scaffoldprohead core protein	177	1020	+
gp23 precursor of major head subunit	176	1359	+
gp25 base plate wedge subunit	210	414	+
gp26 baseplate hub subunit	69	702	+
gp32 ssDNA binding protein	75	942	+
gp33 late promoter transcription factor	77	246	+
gp41 DNA primase-helicase	145	1383	+
gp43 DNA polymerase	148	2490	+
gp44 clamp loader subunit	159	945	+
gp45 sliding clamp DNA polymerase accessory protein	160	738	+

gp46 recombination endonuclease subunit	166	1716	+
gp47 recombination endonuclease subunit	168	1029	+
gp48 baseplate tail tube cap	73	1014	-
gp53 base plate wedge component	74	879	+
gp55 Sigma factor for late transcription	170	480	+
gp61 DNA primase subunit	132	831	+
gp62 clamp loader subunit	155	396	+
DexA exonuclease A	78	645	+
NrdA ribonucleotide reductase A subunit	131	2310	+
NrdB ribonucleotide reductase B subunit	129	1137	+
NrdC Aerobic thioredoxin	89	252	+
RegA translational repressor of early genes	154	417	+
Td thymidilate synthase	82	684	+
UvsW RNA-DNA + DNA-DNA helicase	172	1464	+
PsbA photosystem II D1 protein	108	1080	+
MazG pyrophosphatase	144	651	+
PhoH P-starvation inducible protein	79	753	+
Hsp20 small heat shock protein	152	447	+
Hli03 high-light inducible protein	114	195	-
CobS porphyrin biosynthetic protein	162	1092	+
Virion structural protein	65	1611	-
Hyp. with DUF1825 domain	76	363	+
Hyp. with carboxypeptidase domain	163	2157	-
Hyp. with CTP transerase domain	140	1089	-
Hyp. with Methylamine utilization domain	171	444	+
Hyp. with Phytanoyl-CoA-dioxygenase domain	32	507	+
Hypothetical protein T4-GC#443	87	240	-

Hypothetical protein T4-GC#176	150	327	-
Hypothetical protein T4-GC#201	133	435	-
Hypothetical protein T4-GC#313	85	432	-
Hypothetical protein T4-GC#321	80	231	-
Hypothetical protein T4-GC#49	164	237	-
Hypothetical protein T4-GC#71	141	1194	-
Hypothetical protein T4-GC#112	202	249	-
Hypothetical protein T4-GC#142	167	261	-
Hypothetical protein T4-GC#152	161	303	+
Hypothetical protein T4-GC#250	116	237	-
Hypothetical protein T4-GC#198	135	372	-
Hypothetical protein T4-GC#43	208	19860	+
dCMP deaminase	-	-	-
Dda DNA helicase	-	-	-
DsbA dsDNA binding protein, late transcription	-	-	-
gp1 dNMP kinase	-	-	-
gp7 baseplate wedge initiator	-	-	-
gp9 baseplate wedge tail fiber connector	206	1497	+
gp10 baseplate wedge subunit and tail pin	206	1497	+
gp11 base plate wedge component	-	-	-
gp12 short tail fiber	-	-	-
gp24 precursor of head vertex subunit	176	1359	+
gp30 DNA ligase	-	-	-
gp31 head assembly cochaperone with GroEL	-	-	-
gp34 long tail fiber, proximal subunit	-	-	-
gp49 recombination endonuclease VII	-	-	-
gp52 DNA topoisomerase subunit	-	-	-

gp54 baseplate tail tube initiator	-	-	-
gp59 loader of gp41 DNA helicase	-	-	-
gp60+39 DNA topoisomerase subunit	-	-	-
Hypothetical-Protein T4-GC#1491	-	-	-
Hypothetical with 5' RNA ligase family domain	-	-	-
Hypothetical with DUF1768 domain	-	-	-
NrdC.11 hypothetical protein	-	-	-
NrdD anaerobic NTP reductase large subunit	-	-	-
NrdH glutaredoxin	-	-	-
PseT polynucleotide 5'-kinase and 3'-phosphatase	-	-	-
RIIA-RIIB membrane-associated	-	-	-
RNaseH ribonuclease	86	879	+
RnlA RNA ligase	-	-	-
Tk thymidine kinase	-	-	-
Tk.4 hypothetical protein	-	-	-
Vs.1 hypothetical with transglycosylase SLT domain	-	-	-
Wac fibritin neck whiskers	-	-	-

Table S3. tRNA genes predicted in the genome of cyanophage S-SZBM1.

tRNA no.	Begin	End	tRNA type	Anti codon	Inf Score	Sequence
1	68288	68215	Arg	TCT	74.00	GTCTCAGTAGCTCAGTTGGATAGAGCATCTGCCTTCTAAGCAGTTGGTCGGGGGTTCGAGTCCCTCCTGAGACG
2	68211	68140	Val	TAC	75.70	GGGAGATTAGCTCAGCGGTAGAGCAATTGCCTTACAAGCAATGGGTCACTGGTTCGATCCCAGTATCTCCCA
3	31939	31866	Thr	TGT	73.00	GCCTGAGTAGCTCAGCTGGATAGAGCAACGGTTTTGTAAACCGTAGGTCGTCGGTTCGAGTCCGACCTTAGGCT
4	31761	31688	Pro	TGG	73.10	CGAAGCGTAGCGCAGTTTGGTAGCGCACTGCTTTTGGGAAGCAGGGGTCGTAGGTTCGATTCCTACCGTTTCGA
5	31405	31333	Ala	TGC	66.70	GGGGAATTAGCTCATTTGGTAGAGCGCCTGCTTTGCACGCAGGAGGTAAGGGGTTCGACTCCCCTATTCTCCA
6	31284	31213	Asn	GTT	71.30	TCCTGCTTAGCTCAGCGGTAGAGCGAACGACTGTTAATCGTTTGGTCCCTGGTTCGATCCCAGGAGCAGGAG

Table S4. Homologs of cyanophage S-SZBM1 predicted in the non-redundant database base on the complete nucleotide sequence.

Scientific Name	Max Score	Total Score	Query Cover	*e*-value	Identity	Accession
Synechococcus phage S-WAM1	3659	35918	38.00%	0	78.95%	KU686210.1
Prochlorococcus phage P-RSM6	2686	10318	12.00%	0	78.60%	HQ634193.1
Prochlorococcus phage P-TIM40	1666	4410	6.00%	0	74.58%	KP211958.1
Synechococcus phage S-H25	1369	5728	5.00%	0	80.72%	MT162468.1
Synechococcus phage S-H38	1336	4575	3.00%	0	85.49%	MW117965.1
Synechococcus phage S-RIM2	1280	4463	3.00%	0	79.85%	KX349234.1
Synechococcus phage S-RIM12	1245	3388	3.00%	0	80.85%	KX349307.1
Synechococcus phage S-T4	1221	2325	2.00%	0	79.28%	NC_048049.1
Synechococcus phage S-ShM2	1194	2030	2.00%	0	76.90%	GU071096.1
Synechococcus phage S-RIM14	1194	2030	2.00%	0	76.87%	KX349304.1
Synechococcus phage S-SSM2	1188	2125	2.00%	0	76.85%	JF974292.1
Synechococcus virus S-PRM1	1157	2851	2.00%	0	78.54%	NC_055761.1
Synechococcus phage S-PM2	1110	4179	3.00%	0	84.92%	AJ630128.1
Prochlorococcus phage Syn33	1134	3277	3.00%	0	79.67%	GU071108.1
